# Prevalence and Risk Factors for Hearing Loss in Neonates Admitted to the Neonatal Intensive Care Unit: A Hospital Study

**DOI:** 10.7759/cureus.11207

**Published:** 2020-10-27

**Authors:** Amir Kamal Hardani, Elena Goodarzi, Maryam Delphi, Gholamreza Badfar

**Affiliations:** 1 Department of Pediatrics, Abuzar Children's Hospital, Ahvaz Jundishapur University of Medical Sciences, Ahvaz, IRN; 2 Department of Audiology, Ahvaz Jundishapur University of Medical Sciences, Ahvaz, IRN; 3 Department of Pediatrics, Ahvaz Jundishapur University of Medical Sciences, Ahvaz, IRN

**Keywords:** neonate, risk factors, hearing screening, transient evoked otoacoustic emissions, automated auditory brain stem response

## Abstract

Introduction: Hearing loss is one of the most common congenital disabilities in neonates. The aims of this study were to investigate the prevalence of hearing loss and identify the most significant risk factor in neonates hospitalized at the Neonatal Intensive Care Unit (NICU).

Methods: This cross-sectional study involved 530 neonates admitted to NICU Abuzar Hospital with risk factors for hearing loss based on Joint Committee of Infant Hearing (JCIH). The hearing screening tests include transient evoked otoacoustic emissions (TEOAES) and the automated auditory brain stem response (AABR). For infants with abnormal AABR and TEOAE results, the Auditory Brainstem Response (ABR) and Auditory Steady-State Responses (ASSR) tests were performed.

Result: Of 530 infants, 27 (5.09%) were diagnosed with different types of hearing loss. Ototoxic drugs, hyperbilirubinemia requiring exchange transfusion, asphyxia, low weight birth, Apgar score < 5, and a kinship marriage of parents were significant risk factors for hearing loss in our study population.

Conclusion: Due to the high prevalence of hearing loss in the NICU, it is recommended that a hearing screening program be performed for all infants admitted to the NICU. Implement a comprehensive plan for neonatal hearing screening for early detection and intervention of hearing loss is essential.

## Introduction

Early screening and detection of hearing loss in infants and children to intervene and reduce the adverse effects of hearing loss are essential. Delay in the process of diagnostic hearing loss will lead to adverse consequences on the development of the child's speech-language and cognitive skills [1].

More than 80% of hearing loss in children is congenital or occurs in infancy. Bilateral deafness is reported in 1-3% of births and in 2-4% of neonates in the intensive care unit, which is more common than disorders such as congenital hypothyroidism and phenylketonuria [2]. The frequency of hearing loss in high-risk infants is 10-20 times [3] and sometimes even 10-50 times [4] higher than infants without risk factors for hearing loss.

In 1994, 2000, and 2007, the Joint Committee of Infant Hearing (JCIH) published the risk factors for hearing loss and set standards for the identification of hearing loss [5].

However, it requires many studies to investigate the effect of risk factors on hearing loss incidence. These factors include: birth weight less than 1250 grams, prematurity, hypoxia, Apgar score less than 3 and 6 in one and five minutes respectively, seizures, ventilation, meningitis, jaundice, asphyxia, hypoglycemia, and treatment with aminoglycosides and furosemide [5]. It is noting that hearing loss and brainstem disorder are more common in infants admitted to the Neonatal Intensive Care Unit (NICU).

Many organizations emphasize the need for a comprehensive neonatal hearing screening program at birth and before hospital discharge. Until 1990, neonatal hearing screening was performed only in high-risk neonates, which resulted in detection of 50% of infants with hearing loss. In 2000, the Joint Committee of Infant Hearing recommended that all infants participate in the screening program [6].

According to the Joint Committee of Infant Hearing, The Automated Auditory Brainstem Response (AABR) and Transient Evoked Otoacoustic Emission (TEOAE) tests are used to screen children's hearing. Otoacoustic emission is a non-invasive, rapid, and appropriate method for assessing cochlear function. However, the diagnostic value of TEOAE alone is limited and ABR is more reliable for hearing screening [7].

Early detection of hearing loss in the infant is an essential step in preventing family, social, welfare, speech, language and cognitive problems. The importance of early hearing screening has been recognized for many years and follows two primary goals. Its short-term goal is early detection of hearing loss and its long-term goal is to improve speech, language, and cognitive development [8].

We aimed to determine the prevalence of hearing loss and the associated risk factors in NICUs of Abuzar hospitals, affiliated to Ahwaz University of Medical Sciences.

## Materials and methods

The study was a cross-sectional descriptive study of newborns admitted into the NICU of the Aboozar Children’s Hospital (Jundishapur University of Medical Sciences), Ahvaz, Iran, between August 2019 and April 2020. Newborns whose parents gave written informed consent were included in the study. The Ethics Deputy of Ahvaz Jundishapur University of Medical Sciences provided IRB# IR.AJUMS.REC.1399.240.

Five hundred thirty neonates in the NICU of Abuzar Hospital in Ahvaz via non-random convenience sampling were selected and screened based on the Welfare Organization of Iran’s protocol in parallel method. In this method, Oto-Acoustic Emission (OAE) and AABR hearing tests are performed simultaneously in one day.

All enrolled neonates were screened for hearing impairment with TEOAE and AABR by Madsen Accuscreen (Madsen; Natus Hearing & Balance (formerly Otometrics); Taastrup, Denmark). Audiologists performed all tests. For TEOAE click stimulus was used at the intensity of 80 dB sound pressure level (SPL). The time required for the test was about one to three minutes. The newborns were tested in a quiet (preferably in sleep or while breastfeeding) room. The pass criterion for TEOAE was 6 dB signal to noise ratio at least in three frequencies from 1000 to 4000 HZ. AABR was conducted by click stimuli at 35 dBnHL, with alternating polarity and presentation rate of 37.7/s. The AABR electrode arrangement was as follows: the negative electrode was placed on the ipsilateral mastoid, the ground electrode on the contralateral mastoid and the positive electrode on the vertex. Neonates who had abnormal results in either test or either ear were reported as fail. Neonates with fail results were tested after two weeks. Before the second screening, high frequency tympanometry was performed, and neonates with middle ear effusion were referred for medical treatment. Impedance Audiometer AZ 26 (Interacoustics, Eden Prairie, MN, USA) was used to record the immittance measures. A high-frequency probe tone of 1,000 Hz was utilized to measure Y-admittance tympanograms, with a positive to negative pressure sweep of 200 dapa at a pump speed of 50 dapa/s [9]. In the second screening, TEOAE and AABR were re-tested in neonates with normal middle ear function. Neonates with middle ear effusions were re-tested after treatment. Neonates with hearing-impairment risk factors according to JCIH risk factors [10] and pass results in second screening will be re-tested before six, nine, 18 and 30 months of age. Neonates with fail results in the second screening were referred for diagnostic ABR (Charter EP200; ICS) and ASSR (Charter EP200; ICS) for determining the degree of hearing loss and based on the results rehabilitation process started. The age of children referred to didnostic ABR&ASSR was between three to six months. Diagnostic ABR was performed by click stimuli, presentation rate of 21.1/s with rarefaction polarity. Rarefaction polarity has better and more stable results of ABR tests [11]. Wave V was traced by decreasing intensity for determining the auditory threshold. ASSR was performed for octave frequencies from 500 to 4000 HZ simultaneously for both ears.

In this study, association between hearing loss and risk factors such as birth weight <1500 g, family history of hearing loss, kinship marriage of parents, hyperbilirubinemia at serum levels requiring exchange transfusion, prematurity, mechanical ventilation >5 days, asphyxia, any syndrome, ototoxic drug, sepsis, hypoglycemia, and Apgar score <5 at one minute after birth was evaluated.

Finally, all information was coded in Statistical Package for Social Sciences (SPSS) Statistics version 22 (IBM Corp., Armonk, NY, USA) software and analyzed at the significance level of 0.05. Frequency distribution for descriptive statistics and Chi-square test to evaluate the association between hearing loss and risk factors were used.

## Results

In this cross-sectional study, 51.32% of the subjects were females and 48.67% were males. The neonates’ mean age was 6.65 ± 6.96 days (age range: 0 - 40 days).

There were 530 NICU infants who underwent AABR and TEOAE tests. Of these, 78 (14.71%) infants failed in the first screening and entered the second screening. In the second screening, 28 (5.28%) of 78 infants failed and were referred for diagnostic ABR and ASSR. Three children were missing from the study due to non-referral to the second screening (Table 1).

**Table 1:** The result of first and second screening tests

Auditory test	fail	pass	missing
First screening	78(14.71%)	452(85.29%)	---
Second screening	28(5.28%)	47(8.86%)	3(0.56%)

According to diagnostic tests, there were 27 (5.09%) infants with hearing loss, 14 cases with SNHL, seven with conductive hearing loss, and six with auditory neuropathy. At the diagnostic stage, OAE test was performed for all infants. One infant due to non-referral to the diagnostic test was missed.The percentage of hearing loss in each type of hearing loss is given in Table 2.

**Table 2:** The result of diagnostic hearing test

Type of hearing loss	missing	SNHL	CHL	neuropathy
ABR,ASSR	1(0.18%)	14(2.64%)	7(1.32%)	6(1.13%)

Results of Chi-square test revealed no significant association between hearing loss and infants’ age (P = 0.620), sex (P = 0.126), prematurity (P = 0.901), ventilation (P = 0.069), sepsis (P = 0.086), syndrome (P = 0.446), hypoglycemia (P = 0.679) and family history of hearing loss (P = 0.067). However, there was a statistically significant association between hearing loss and birth weight (P < 0.001), Apgar score < 5 (P < 0.001), hyperbilirubinemia (P < 0.001), ototoxic drug (P=0.050), asphyxia (P = 0.041) and kinship marriage of parents (P = 0.036) (Table 3).

**Table 3:** The relationship between the prevalence of hearing loss and risk factor P-value < 0.05 was considered statistically significant.

factor	infant without hearing loss(n)	Infant with hearing loss(n)	P-value
Sex Male	227	16	0.126
Female	272	11	
Birth weight less than 1500 g Yes	78	18	< 0.001*
No	421	9	
Family history of hearing loss Yes	20	9	0.067
No	479	18	
Kinship marriage of parents Yes	91	12	0.036*
No	408	15	
Hyperbilirubinemia Yes	385	18	< 0.001*
No	114	9	
Prematurity Yes	270	15	0.901
No	229	12	
Ototoxic drug Yes	399	10	0.050*
No	100	17	
Ventilation>5days Yes	288	11	0.069
No	211	16	
Apgar Score <5	126	18	< 0.001*
>5	373	9	
Sepsis Yes	283	17	0.086
No	216	10	
Syndrome Yes	2	1	0.446
No	497	26	
Hypoglycemia Yes	208	10	0.679
No	291	17	
Asphyxia Yes	187	15	0.041*
No	312	12	

## Discussion

Hearing screening is the primary method for early detection of hearing loss. The procedure should be simple and fast. The NICU graduate has a higher risk of developing hearing loss than the general population.

In the current study, based on the final ABR, ASSR results, 27 cases (5.09%) had hearing loss. Molini et al. reported a prevalence of 4.3% for infants at risk of hearing loss [12] is close to the prevalence rate reported in the present study. Also, in studies by Ohl in 2009 and Taghdiri in 2008, 4.55% and 4% of the newborns were affected by hearing loss, respectively [4,13]. In Rai and Thakurs’ study, the prevalence of hearing loss in infants admitted to the intensive care unit was 4.91% (49.18 per 1000 live births) [14]. These findings are similar to our results.

Hille et al. reported a prevalence of 3.2% in NICU infants [15], which is lower the prevalence rate reported in the present study.

In a study by Pourarian in 2012, 13.7% of infants in NICU suffered from hearing loss, which was higher than the expected rate [16]. This discrepancy in the prevalence of hearing loss might be related to differences in screening and diagnostic of hearing methods. In our study, a parallel method was used for hearing screening and ABR&ASSR test for hearing loss diagnosis.

According to the diagnostic test, there were 5.09% infants with hearing loss, 2.64% with SNHL, 1.32% with conductive hearing loss, and 1.13% with auditory neuropathy. In a study by Colella in 2014, the incidence of hearing loss in the sample was approximately 3%, of which 0.91% was sensorineural, 0.13% with auditory neuropathy spectrum disorder (ANSD), and 1.8% conductive [6].

The probability of hearing loss increases with an increase in the number of risk factors [5]. In our study, 96% of infants with hearing loss had three or more risk factors. In Jayagobi's study, 38% of infants with SNHL, mixed hearing loss, and auditory neuropathy/auditory dyssynchrony (AN/AD) had four or more risk factors [17]. In this study, the hearing loss risk factors included hyperbilirubinemia requiring exchange transfusion, low birth weight (<1500 g), ototoxic drug, Apgar score <5, asphyxia, and kinship marriage of parents. In a study by Ohl, the risk factors for hearing loss included neurological disorders, asphyxia, family history of hearing loss, and TORCH (toxoplasmosis, other agents, rubella, cytomegalovirus, herpes simplex) infections; however, hearing loss was not asso­ciated with birth weight below 1500 g or birth before 34 weeks of gestation [4].

According to Recchia et al., the use of ototoxic drugs is one of the causes of hearing loss in infants hospitalized in the NICU [18]. In our study, the association between hearing loss and antibiotic therapy was also significant, which is consistent with their study.

Also, in a study by Amini, a significant relationship was found between the mean birth weight and hearing loss; however, no statistical cor­relation was found between Apgar score and abnormal OAE [19] but, In the present study Apgar <5 and low weight birth were high-risk factors for hearing loss.

The prevalence of hearing loss due to positive history in infants is 7.29% [20]. In our study, no significant correlation was found between family history and hearing loss. However this is an established risk factor for congenital hearing loss, this discrepancy may be because of sample size and did not reflect congenital type hearing loss in family history. 

According to previous studies, one of the most critical risk factors for hearing loss is the high bilirubin level during the neonatal period. In a study by Boo in 2008, 32 neonates (8.12%) presented with severe hyperbilirubinemia and unilateral or bilat­eral permanent hearing loss [21]. In the present study, there was a high association between hyperbilirubinemia and hearing loss.

In the study conducted by Khairy in 2017, sepsis was the risk factor for hearing loss in infants [7], but in the Alaee study, no correlation was found between sepsis and hearing loss [22]. This result is consistent with our findings.

One of the risk factors studied in the present study was ventilation. A study by Gohari showed no significant relationship between a ventilation risk factor and hearing loss [23]. This finding is in line with our results.

In the present study, prematurity was investigated as one of the risk factors for hearing loss. Chi-square test revealed no correlation between this factor and hearing loss. Most premature infants who failed the first screening test showed normal hearing in the final audiologic tests. In addition, several studies reported that auditory maturation can affect the long-term hearing result of prematurity [24].

Another definitive finding of this study is the effect of the kinship marriage of parents on the incidence of hearing loss. These findings have been less attention in previous studies. In the study of Gohari no significant correlation was found between kinship marriages of parents and hearing loss [23]. In the Khuzestan province, due to the cultural context, the kinship marriage of parents is one of the risk factors for hearing loss.

Literature studies also found a significant relationship between hearing loss and neonatal asphyxia associated with hearing loss [6], like our findings.

It is essential to consider the risk factors of infants admitted in the NICU, not to select candidates for hearing screening, but to the most appropriate management and follow-up for each case. Hearing screening, diagnosis, and follow-up of hearing loss in risk factor infants are necessary steps of the hearing child health program. At the same time, keep in mind that targeted screening of only high-risk criteria for hearing loss may miss up to 50% of infants with hearing loss. Hence, in 1993 a National Institutes of Health Consensus Statement recommended Universal Newborn Hearing Screening (UNHS). Screening before one month of age, detecting hearing loss before three months of age, and intervention services initiated by six months of age are recommended.

The strengths of the present study combination of both TEOAEs and AABR, as it can diagnose auditory neuropathy, use of one single measurement tool, and performance of evalua­tions by one audiologist, which could prevent mea­surement errors and observer biases.

The limitations of this study was the lack of infants referred to a second screening and diagnostic test.We tried to minimize this limitation by calling and following up the families.

## Conclusions

It can be concluded that the prevalence of hearing loss is high among our high risk neonates and the use of TEOAE and AABR tests simultaneously is essential for these infants. The use of ototoxic drugs, hyperbilirubinemia requiring exchange transfusion, asphyxia, low weight birth, Apgar score < 5 and a Kinship marriage of parents were significant risk factors for hearing loss in our study population. Therefore, timely detection and prevention of the mentioned risk factors are highly recommended.
